# Supplementary material for: A new phased assembly of the Antarctic spiny plunderfish provides novel insights into the evolution of the notothenioid radiation
Source: bioRxiv. 2026 Apr 23:2026.04.21.719633. Preprint. [Version 1] doi: 10.64898/2026.04.21.719633 (PMC13131655; doi:10.64898/2026.04.21.719633)
Supplement: Supplement 1 [file media-1.pdf]

Supplementary Tables

Supplementary Table 1

	Hap1	Hap2
**Total length**	1.068.962.898	1.220.510.434
**Scaff number**	1.435	653
**Contig N50**	17.034.000	12.766.000
**Scaf N50**	46.795.402	42.703.524
**Number of Chromosomes**	24	24
**BUSCO**	99.0%[S:98.4%,D:0.6%],F:0.6%,M:0.4%,n:3640	99.0%[S:98.3%,D:0.7%],F:0.7%,M:0.3%,n:3640
**QV**	40	40,4

Supplementary Table 2

Scaffold	Chromosome
SUPER_4	1
SUPER_24	2
SUPER_2	3
SUPER_17	4
SUPER_12	5
SUPER_8	6
SUPER_3	7
SUPER_13	8
SUPER_1	9
SUPER_9	10
SUPER_16	11
SUPER_20	12
SUPER_6	13
SUPER_18	14
SUPER_7	15
SUPER_5	16
SUPER_10	17
SUPER_23	18
SUPER_21	19
SUPER_22	20
SUPER_11	21
SUPER_14	22
SUPER_19	23
SUPER_15	24

Supplementary Table 3

	Hap1	Hap2
**Number of genes**	23,057	22,463
**Number of mRNAs**	48,897	31,527
**Mean number of introns per mRNA**	8.3	8.4
**Mean number of exons per mRNA**	9.3	9.4
**Mean gene length**	14,904	14,715
**Mean intron length**	1,555	1,522
**Mean exon length**	174	124
**BUSCO (actinopterygii odb_10)**	C:98.9%[S:97.7%,D:1.2%],F:0.5%,M:0.6%	C:96.7%[S:95.6%,D:1.1%],F:1.1%,M:2.2%

Supplementary Table 4

Overview table		
**TE class**	**Hap1**	**Hap2**
**LINEs**	13	11,34
**LTRs**	13,98	17,84
**DNA**	13,88	12,25
**RC**	0,05	0,04
**SINE**	0,06	0,05
**Unknown**	0,2	0,18
**Tandem repeats**	4,5	4,8
**Simple repeats**	2,35	2,07
**Low complexity**	0,15	0,14
**Total**	43,67	43,91

TEs by Superfamily	Genome coverage (%)		
**Superfamily**	**Hap1**	**Hap2**	**Class**
**TcMar-ISRm11**	0,004779691	0,004406599	DNA
**Sola-1**	0,005902255	0,005421994	DNA
**MULE-NOF**	0,006541181	0,006372416	DNA
**R2-NeSL**	0,009075744	0,008463754	LINE
**hAT-hATx**	0,010835457	0,00946604	DNA
**I-Jockey**	0,012170373	0,010227934	LINE
**RTE-X**	0,013299111	0,013012916	LINE
**TcMar-Fot1**	0,01331492	0,011887567	DNA
**hAT-Tag1**	0,016618907	0,014290496	DNA
**TcMar-Tc2**	0,01901053	0,015485161	DNA
**CR1**	0,019605302	0,017100141	LINE
**hAT-hAT5**	0,020206248	0,017028122	DNA
**Zisupton**	0,030205956	0,026711119	DNA
**Penelope**	0,043723406	0,041617096	Penelope

**Helitron**	0,045550098	0,041057248	RC
**Dada**	0,04571287	0,062060018	DNA
**PIF-ISL2EU**	0,053272965	0,046735528	DNA
**Unknown**	0,05527562	0,046773218	SINE
**Merlin**	0,055644008	0,04861294	DNA
**Proto2**	0,056050657	0,047631055	LINE
**Crypton-H**	0,065469906	0,05653356	DNA
**Ginger-1**	0,077340741	0,06877434	DNA
**Ngaro**	0,079720203	0,071085996	LTR
**Crypton-A**	0,088138124	0,076345271	DNA
**PIF-Harbinger**	0,097676365	0,077930264	DNA
**Copia**	0,098900895	0,085675957	LTR
**Sola-2**	0,099819246	0,086373289	DNA
**IS3EU**	0,126678184	0,110161533	DNA
**hAT-Blackjack**	0,128556888	0,113959288	DNA
**Kolobok-E**	0,147513816	0,319307471	DNA
**CMC-EnSpm**	0,148790452	0,136953848	DNA
**ERV**	0,15933582	0,141425665	LTR
**Unknown**	0,20210991	0,177821585	Unknown
**Maverick**	0,239393727	0,207085079	DNA
**P**	0,250950338	0,218685472	DNA
**Unknown**	0,326903964	0,27877443	LINE
**L1-Tx1**	0,337941388	0,26045185	LINE
**L1**	0,343850379	0,316897578	LINE
**Pao**	0,346597293	0,293703266	LTR
**Kolobok-T2**	0,364417532	0,319307471	DNA
**PiggyBac**	0,51869938	0,448819842	DNA
**hAT-Tip100**	0,554194204	0,484122203	DNA
**RTE-BovB**	0,612075295	0,542266565	LINE
**DIRS**	0,660656504	0,579024546	LTR
**I**	0,684329604	0,577757126	LINE
**hAT-Charlie**	0,685612134	0,610916039	DNA
**TcMar-Tc1**	0,751543413	0,662926983	DNA
**Rex-Babar**	0,919560583	0,788629637	LINE
**hAT-Ac**	1,029050438	0,903821606	DNA

**ERV1**	1,093929033	0,957094071	LTR
**Gypsy**	1,999559437	1,72982667	LTR
**Unknown**	8,221667769	7,274402621	DNA
**Unknown**	9,539620475	13,98632656	LTR
**L2**	9,630117667	8,437966619	LINE

Supplementary Table 5a

Species	NCBI taxon ID	Assembly ID	Family	size (Mb)	Assembly size	Contig N50 (Mb)	Scaffold N50 (Mb)	BUSCO	Assembly Accession	Reference
Cottoperca gobio (Cottoperca trigloides)	56716	fCotGob3.1	Bovichtidae	609	609.4	6,3	25,2	C:93.9%[S:92.5%,D:1.4%],F:0.9%,M:5.2%	GCF_900634415.1	Bista et al. 2020
Eleginops maclovinus	56733	JC_Emac_rtc_rv5	Eleginopsidae	606	606.3	7,6	26,7	C:98.0%[S:97.0%,D:1.0%],F:0.4%,M:1.6%	GCF_036324505.1	Cheng et al. 2024
Dissostichus mawsonii	36200	KU_Dm_1.0	Nototheniidae / Pleuragrammatinae	926	926.4	3	37	C:95.6%[S:92.1%,D:3.5%],F:1.7%,M:2.7%	GCA_011823955.1	Lee at al., 2021
Dissostichus eleginoides	100907	KU_De_1.0	Nototheniidae / Pleuragrammatinae	843	844.7	4,2	36	C:97.8%[S:94.7%,D:3.1%],F:1.0%,M:1.2%	GCA_031216635.1	Lee et al. 2023
Pagothenia borchgrevinki (Trematomus borchgrevinki)	8213	PborFfbFv9	Nototheniidae / Trematominae	935	935.1	1,8	42,7	C:98.0%[S:96.4%,D:1.6%],F:0.7%,M:1.3%	GCA_044885175.1	Rayamajhi et al. 2025
Notothenia rossii	101497	fNotRos5.1	Nototheniidae / Nototheniinae	1000	1,043	0,383	89,7	C:94.8%[S:94.0%,D:0.8%],F:2.0%,M:3.2%	GCA_949606895.1	Bista et al. 2024
Harpagifer antarcticus	43256	fHarAnt1.2	Harpagiferidae	1000	1.072 / 1.222	17	44,8	C:99.0%[S:98.4%,D:0.6%],F:0.6%,M:0.4%	tbc	this manuscript
Pogonophryne albipinna	1090488	KU_S6	Artedidraconidae	1100	1.074	0,962	41,8	C:97.0%[S:94.3%,D:2.7%],F:0.9%,M:2.1%	GCA_028583405.1	Jo et al. 2023
Pseudochaenichthys georgianus	52239	fPseGeo1.2	Channichthyidae	1100	1.026	0,657	42,8	C:94.1%[S:93.0%,D:1.1%],F:1.3%,M:4.6%	GCF_902827115.2	Bista et al., 2023

Supplementary Table 5b

Species	NCBI taxon ID	Assembly ID	Family	size (Mb)	Assembly size	Contig N50 (Mb)	Scaffold N50 (Mb)	BUSCO	Assembly Accession	Reference
Champsocephalus gunnari	52237	JC_Cgun_ftc_fv8	Channichthyidae	994	994.2	3,2	44,1	C:97.8%[S:96.2%,D:1.6%],F:0.7%,M:1.5%	GCA_036324595.1	River-Colon et al., 2023
Champsocephalus esox	159716	JC_Ceso_ftc_fv8	Channichthyidae	987	987.1	2,6	43,6	C:97.3%[S:96.2%,D:1.1%],F:1.0%,M:1.7%	GCA_036324585.1	River-Colon et al., 2023
Chaenocephalus aceratus	36190	KU_Ca_2.0	Channichthyidae	1100	1.065	1,5	33,5	C:92.6%[S:88.9%,D:3.7%],F:1.5%,M:5.9%	GCA_023974075.1	Lee et al., 2023
Gasterosteus aculeatus	481459	GAculeatus_UGA_version5	Gasterosteidae	472	471.9	0.4858	20.4	C:97.0%[S:94.6%,D:2.4%],F:1.1%,M:1.9%	GCF_016920845.1	Nath et al., 2021
Pungitius pungitius	134920	fPunPun2.1	Gasterosteidae	480	480.4	1.4	21	C:97.8%[S:97.0%,D:0.8%],F:0.8%,M:1.4%	GCF_949316345.1	Hänfling et al., 2023
Larimichthys crocea	215358	L_crocea_2.0	Sciaenidae	658	657.9	0.2775	27	C:98.4%[S:97.5%,D:0.9%],F:0.5%,M:1.1%	GCF_000972845.2	Ao et al., 2015
Labrus bergylta	56723	fLabBer1.1	Labridae	720	720.2	2.7	31.3	C:97.8%[S:97.1%,D:0.7%],F:0.6%,M:1.6%	GCF_963930695.1	NA
Notolabrus celidotus	1203425	fNotCel1.pri	Labridae	847	846.7	3.7	37.1	C:95.8%[S:94.9%,D:0.9%],F:0.6%,M:3.6%	GCF_009762535.1	NA
Nelusetta ayraudi	303726	CSIRO-AGI_Nayr_v1	Monacanthidae	577	576.5	6.9	6.9	C:96.7%[S:95.7%,D:1.0%],F:1.0%,M:2.3%	GCF_046127955.1	NA
Takifugu rubripes	31033	fTakRub1.2	Tetraodontidae	384	384.1	3.1	16.7	C:96.9%[S:94.3%,D:2.6%],F:0.7%,M:2.4%	GCF_901000725.2	NA

Supplementary Table 6

Variant type	Number of events	Length reference	Length query
**Inversions**	155	8.868.241	9.134.047
**Translocations**	145	4.197.546	3.647.854
**Duplications**	251	4.609.203	-
**Insertions**	214.681		16.640.238
**Deletions**	214.893	16.808.012	-
**SNPs**	1.620.216	52.762.169	50.471.908
**Highly diverged regions**	24.540	14.694	14.694

Supplementary Table 7

	Split	Deletions (bp)	Insertions (bp)	Net gain (Mb)	Branch length (MY)	Net gain rate (Mb X MY)
**Anc01**	EleMac - cryonotothenioids	499043	100799664	100,30	20.7	4.84544062801932
**Anc02**	Cryonotothenioid ancestor	5942195	262680582	256,74	15.6	16.4575889102564
**Anc03**	DisMaw - DisEle	1915618	98933047	97,02	4.65	20.8639632258065
**Anc04**	PagBor - NotRos	944905	20853349	19,91	1.2	16.59037
**Anc05**	NotRos - PogAlb	7118060	22274394	15,16	1.48	10.2407662162162
**Anc06**	PogAlb - ChaGun	7995185	59483192	51,49	1.87	27.5336935828877
**Anc07**	PogAlb - HarAnt	3246574	53989080	50,74	1.2	42.2854216666667
**Anc08**	ChaGun - PseGeo	6099811	111093996	104,99	2.12	49.5255589622641
**Anc09**	ChaGun - ChaEso	4932407	163657480	158,73	2.09	75.9450110047847
**Anc10**	PseGeo - ChaAce	2868300	62970793	60,10	0.79	76.0791050632911
**ChaAce**	/	5511882	147085816	141,57	3.24	43.6956586419753
**ChaEso**	/	6638005	106860420	100,22	1.93	51.9287124352332
**ChaGun**	/	3755646	99118512	95,36	1.93	49.4108113989637
**CotGob**	/	5227	187530812	187,53	47	3.98990606382979
**DisEle**	/	4580041	87105609	82,53	6.05	13,6405897520661
**DisMaw**	/	4639833	132781946	128,14	6.05	21,1805145454545
**EleMac**	/	8496818	139792732	131,30	26.3	4.99224007604563
**HarAnt**	/	10392579	351876096	341,48	4.95	68.9865690909091
**NotRos**	/	10414889	399848994	389,43	8.01	48.6184900124844
**PagBor**	/	13108365	278777704	265,67	9.5	27.9651935789474
**PogAlb**	/	5204039	240104298	234,90	4.95	47.4545977777778
**PseGeo**	/	5467138	217171889	211,70	3.24	65.3409725308642

Supplementary Table 8

Node	Split	DNA	LINE	LTR	Total	Branch length	Rate x MY
**Anc01**	EleMac - cryonotothenioids	3342	1469	1173	5984	20,7	289,0821256
**Anc02**	Cryonotothenioid ancestor	9635	9767	3992	23394	15,6	1499,615385
**Anc04**	PagBor - NotRos	1579	991	537	3107	1,2	2589,166667
**Anc05**	NotRos - PogAlb	4320	3092	1315	8727	1,48	5896,621622
**Anc06**	PogAlb - ChaGun	8442	7314	2581	18337	1,87	9805,882353
**Anc07**	PogAlb - HarAnt	4040	2864	1339	8243	1,2	6869,166667
**HarAnt**	/	16994	23600	11163	51757	4,95	10455,9596

Supplementary Table 9

GO.ID	Term	Annotated	Significant	Expected	classicFisher
GO:0060021	roof of mouth development	84	15	4,97	0,00017
GO:0010761	fibroblast migration	38	8	2,25	0,00024
GO:0035019	somatic stem cell population maintenance	56	11	3,31	0,00037
GO:0008542	visual learning	56	11	3,31	0,00037
GO:0045666	positive regulation of neuron differentiation	414	42	24,47	0,00042
GO:0007399	nervous system development	2406	205	142,22	0,00046
GO:0023052	signaling	5150	373	304,41	0,00049
GO:0007267	cell-cell signaling	1389	115	82,1	0,00051
GO:0072176	nephric duct development	19	6	1,12	0,00058
GO:0001655	urogenital system development	381	41	22,52	0,0006
GO:0045777	positive regulation of blood pressure	40	8	2,36	0,00079
GO:0043401	steroid hormone receptor signaling pathway	180	19	10,64	0,00079
GO:0030099	myeloid cell differentiation	407	31	24,06	0,00089
GO:0006367	transcription initiation at RNA polymerase II promoter	203	22	12	0,00095
GO:0042474	middle ear morphogenesis	21	6	1,24	0,00105
GO:0009415	response to water	21	6	1,24	0,00105
GO:0010628	positive regulation of gene expression	1909	140	112,84	0,00112
GO:0050810	regulation of steroid biosynthetic process	82	9	4,85	0,0012
GO:0051965	positive regulation of synapse assembly	64	11	3,78	0,0012
GO:0048384	retinoic acid receptor signaling pathway	33	8	1,95	0,00128
GO:0048865	stem cell fate commitment	15	5	0,89	0,0013
GO:0045987	positive regulation of smooth muscle contraction	38	8	2,25	0,00144

GO:0009952	anterior/posterior pattern specification	261	29	15,43	0,00168
GO:0010718	positive regulation of epithelial to mesenchymal transition	39	8	2,31	0,00172
GO:0033574	response to testosterone	67	11	3,96	0,00174
GO:0035850	epithelial cell differentiation involved in kidney development	48	9	2,84	0,00175
GO:0051354	negative regulation of oxidoreductase activity	23	6	1,36	0,00177
GO:0050869	negative regulation of B cell activation	34	6	2,01	0,0018
GO:0030326	embryonic limb morphogenesis	134	15	7,92	0,00182
GO:0050769	positive regulation of neurogenesis	510	47	30,15	0,00186
GO:1902305	regulation of sodium ion transmembrane transport	53	7	3,13	0,00187
GO:0030278	regulation of ossification	196	24	11,59	0,00213
GO:0009950	dorsal/ventral axis specification	24	6	1,42	0,00225
GO:0048670	regulation of collateral sprouting	24	6	1,42	0,00225
GO:0003016	respiratory system process	33	6	1,95	0,00243
GO:0001764	neuron migration	173	22	10,23	0,00269
GO:0050919	negative chemotaxis	42	8	2,48	0,00282
GO:0050768	negative regulation of neurogenesis	311	33	18,38	0,00303
GO:0048368	lateral mesoderm development	18	5	1,06	0,0032
GO:0002063	chondrocyte development	34	7	2,01	0,00324
GO:0002062	chondrocyte differentiation	108	16	6,38	0,00336
GO:0060669	embryonic placenta morphogenesis	31	7	1,83	0,00346
GO:0007187	G protein-coupled receptor signaling pathway, coupled to cyclic nucleotide second messenger	187	21	11,05	0,00347
GO:0032330	regulation of chondrocyte differentiation	61	9	3,61	0,00405
GO:0001706	endoderm formation	68	9	4,02	0,00406
GO:0060914	heart formation	27	6	1,6	0,00425
GO:0003151	outflow tract morphogenesis	81	13	4,79	0,00451
GO:0010470	regulation of gastrulation	55	9	3,25	0,00461

GO:0003413	chondrocyte differentiation involved in endochondral bone morphogenesis	20	5	1,18	0,00525
GO:0030857	negative regulation of epithelial cell differentiation	37	7	2,19	0,00532
GO:1902742	apoptotic process involved in development	42	7	2,48	0,00597
GO:0001541	ovarian follicle development	89	12	5,26	0,00598
GO:0030282	bone mineralization	98	14	5,79	0,00605
GO:0048662	negative regulation of smooth muscle cell proliferation	45	7	2,66	0,00616
GO:0021983	pituitary gland development	60	8	3,55	0,00621
GO:0030540	female genitalia development	21	5	1,24	0,00656
GO:0086012	membrane depolarization during cardiac muscle cell action potential	21	5	1,24	0,00656
GO:0048745	smooth muscle tissue development	21	5	1,24	0,00656
GO:0086091	regulation of heart rate by cardiac conduction	39	7	2,31	0,00718
GO:2000772	regulation of cellular senescence	30	6	1,77	0,00732
GO:1902930	regulation of alcohol biosynthetic process	69	7	4,08	0,00753
GO:0033339	pectoral fin development	22	5	1,3	0,00808
GO:0060706	cell differentiation involved in embryonic placenta development	22	5	1,3	0,00808
GO:0001893	maternal placenta development	35	8	2,07	0,00909
GO:0038179	neurotrophin signaling pathway	38	5	2,25	0,0092
GO:0055012	ventricular cardiac muscle cell differentiation	23	4	1,36	0,0092
GO:0060285	cilium-dependent cell motility	74	7	4,37	0,0092
GO:0007156	homophilic cell-cell adhesion	72	10	4,26	0,00945
GO:0009607	response to biotic stimulus	1035	57	61,18	0,00945
GO:0035148	tube formation	177	20	10,46	0,0095
GO:0071229	cellular response to acid chemical	210	19	12,41	0,00967
GO:0060324	face development	63	10	3,72	0,00969
GO:0072102	glomerulus morphogenesis	15	4	0,89	0,00979
GO:0035357	peroxisome proliferator activated receptor signaling pathway	15	4	0,89	0,00979

GO:0039019	pronephric nephron development	15	4	0,89	0,00979
GO:0019371	cyclooxygenase pathway	15	4	0,89	0,00979
GO:0045622	regulation of T-helper cell differentiation	23	5	1,36	0,00983
GO:0072202	cell differentiation involved in metanephros development	23	5	1,36	0,00983
GO:0019395	fatty acid oxidation	99	8	5,85	0,00983
GO:0090189	regulation of branching involved in ureteric bud morphogenesis	25	5	1,48	0,01001
GO:0061217	regulation of mesonephros development	28	5	1,66	0,01002
GO:0014020	primary neural tube formation	102	9	6,03	0,01003
GO:0002684	positive regulation of immune system process	764	46	45,16	0,01008
GO:0000902	cell morphogenesis	1013	75	59,88	0,0102
GO:0032879	regulation of localization	2370	145	140,09	0,01026
GO:0043065	positive regulation of apoptotic process	604	45	35,7	0,01046
GO:0098609	cell-cell adhesion	573	57	33,87	0,01062
GO:2000027	regulation of animal organ morphogenesis	218	21	12,89	0,01102
GO:0007398	ectoderm development	24	5	1,42	0,01183
GO:0048557	embryonic digestive tract morphogenesis	24	5	1,42	0,01183
GO:0030325	adrenal gland development	43	7	2,54	0,01226
GO:0010765	positive regulation of sodium ion transport	38	5	2,25	0,01247
GO:0031667	response to nutrient levels	602	39	35,58	0,01264
GO:0035113	embryonic appendage morphogenesis	143	18	8,45	0,01287
GO:0051896	regulation of phosphatidylinositol 3-kinase/protein kinase B signal transduction	190	16	11,23	0,0131
GO:0043433	negative regulation of DNA-binding transcription factor activity	132	15	7,8	0,01312
GO:0010092	specification of animal organ identity	32	5	1,89	0,01318
GO:0090504	epiboly	46	4	2,72	0,01326

GO:0001708	cell fate specification	112	13	6,62	0,01368
GO:0071880	adenylate cyclase-activating adrenergic receptor signaling pathway	25	5	1,48	0,01408
GO:0060045	positive regulation of cardiac muscle cell proliferation	25	5	1,48	0,01408
GO:0045618	positive regulation of keratinocyte differentiation	17	4	1	0,01553
GO:0060307	regulation of ventricular cardiac muscle cell membrane repolarization	17	4	1	0,01553
GO:1905941	positive regulation of gonad development	17	4	1	0,01553
GO:0072234	metanephric nephron tubule development	17	4	1	0,01553
GO:0031952	regulation of protein autophosphorylation	50	5	2,96	0,0156
GO:0030279	negative regulation of ossification	71	9	4,2	0,01644
GO:0007190	activation of adenylate cyclase activity	26	5	1,54	0,01661
GO:0060009	Sertoli cell development	26	5	1,54	0,01661
GO:0043406	positive regulation of MAP kinase activity	280	29	16,55	0,01699
GO:0048536	spleen development	46	7	2,72	0,0175
GO:0032526	response to retinoic acid	149	18	8,81	0,01752
GO:0030501	positive regulation of bone mineralization	36	6	2,13	0,01781
GO:0019933	cAMP-mediated signaling	143	15	8,45	0,01795
GO:0034105	positive regulation of tissue remodeling	30	5	1,77	0,018
GO:0050673	epithelial cell proliferation	361	36	21,34	0,01806
GO:0021537	telencephalon development	289	31	17,08	0,01835
GO:0051347	obsolete positive regulation of transferase activity	602	53	35,58	0,01858
GO:0045616	regulation of keratinocyte differentiation	35	8	2,07	0,01881
GO:0014912	negative regulation of smooth muscle cell migration	18	4	1,06	0,01906

GO:0061333	renal tubule morphogenesis	82	11	4,85	0,01911
GO:0001657	ureteric bud development	96	12	5,67	0,01912
GO:0002822	regulation of adaptive immune response based on somatic recombination of immune receptors built from immunoglobulin superfamily domains	110	9	6,5	0,01929
GO:0090090	negative regulation of canonical Wnt signaling pathway	116	13	6,86	0,01933
GO:0060444	branching involved in mammary gland duct morphogenesis	27	5	1,6	0,01942
GO:0007413	axonal fasciculation	27	5	1,6	0,01942
GO:0060992	response to fungicide	27	5	1,6	0,01942
GO:0006482	protein demethylation	27	5	1,6	0,01942
GO:0046697	decidualization	27	5	1,6	0,01942
GO:0045665	negative regulation of neuron differentiation	246	23	14,54	0,01975
GO:0048639	positive regulation of developmental growth	184	18	10,88	0,01983
GO:0048169	regulation of long-term neuronal synaptic plasticity	37	6	2,19	0,02022
GO:0001701	in utero embryonic development	372	38	21,99	0,02046
GO:0006469	negative regulation of protein kinase activity	242	22	14,3	0,02057
GO:0010976	positive regulation of neuron projection development	319	28	18,86	0,02204
GO:0043392	negative regulation of DNA binding	50	7	2,96	0,02244
GO:2000378	negative regulation of reactive oxygen species metabolic process	60	7	3,55	0,02251
GO:0051968	positive regulation of synaptic transmission, glutamatergic	28	5	1,66	0,02252
GO:0030890	positive regulation of B cell proliferation	28	5	1,66	0,02252
GO:0086005	ventricular cardiac muscle cell action potential	28	5	1,66	0,02252
GO:0021871	forebrain regionalization	28	5	1,66	0,02252
GO:0021952	central nervous system projection neuron axonogenesis	28	5	1,66	0,02252

GO:0002070	epithelial cell maturation	19	4	1,12	0,02304
GO:0033334	fin morphogenesis	19	4	1,12	0,02304
GO:0048608	reproductive structure development	490	54	28,96	0,02343
GO:0035137	hindlimb morphogenesis	45	8	2,66	0,0235
GO:0055074	calcium ion homeostasis	401	28	23,7	0,02358
GO:0050767	regulation of neurogenesis	847	84	50,07	0,0236
GO:0051146	striated muscle cell differentiation	318	20	18,8	0,02384
GO:0040011	locomotion	1606	123	94,93	0,02416
GO:0050679	positive regulation of epithelial cell proliferation	177	15	10,46	0,02435
GO:0009953	dorsal/ventral pattern formation	142	17	8,39	0,02469
GO:0051674	localization of cell	864	65	51,07	0,02526
GO:0007422	peripheral nervous system development	96	10	5,67	0,02561
GO:0060713	labyrinthine layer morphogenesis	29	5	1,71	0,02593
GO:0031128	developmental induction	43	6	2,54	0,02741
GO:0043550	regulation of lipid kinase activity	62	7	3,66	0,02743
GO:2000737	negative regulation of stem cell differentiation	20	4	1,18	0,02749
GO:0007565	female pregnancy	219	22	12,94	0,02803
GO:0060412	ventricular septum morphogenesis	40	6	2,36	0,02878
GO:0006325	chromatin organization	731	49	43,21	0,02909
GO:0141193	nuclear receptor-mediated signaling pathway	181	22	10,7	0,02936
GO:0045637	regulation of myeloid cell differentiation	212	18	12,53	0,02996
GO:0071805	potassium ion transmembrane transport	187	15	11,05	0,03021
GO:0048659	smooth muscle cell proliferation	147	16	8,69	0,03052
GO:0042633	hair cycle	111	13	6,56	0,03059
GO:0072576	liver morphogenesis	21	4	1,24	0,03088
GO:0003272	endocardial cushion formation	21	3	1,24	0,03095
GO:0032870	cellular response to hormone stimulus	766	67	45,28	0,03196
GO:0007157	heterophilic cell-cell adhesion	41	6	2,42	0,03209

GO:0001702	gastrulation with mouth forming second	41	6	2,42	0,03209
GO:0086014	atrial cardiac muscle cell action potential	21	4	1,24	0,03241
GO:0001889	liver development	222	22	13,12	0,03261
GO:0048863	stem cell differentiation	227	32	13,42	0,0327
GO:0050772	positive regulation of axonogenesis	102	8	6,03	0,0327
GO:0060487	lung epithelial cell differentiation	31	5	1,83	0,03369
GO:0010771	negative regulation of cell morphogenesi...	88	10	5,2	0,03442
GO:0043507	positive regulation of JUN kinase activi...	76	9	4,49	0,03462
GO:0007417	central nervous system development	1117	98	66,02	0,03543
GO:1901570	fatty acid derivative biosynthetic proce...	74	8	4,37	0,03561
GO:0045907	positive regulation of vasoconstriction	42	6	2,48	0,03564
GO:0001756	somitogenesis	101	11	5,97	0,03592
GO:0008284	positive regulation of cell population p...	845	66	49,95	0,03621
GO:0008285	negative regulation of cell population p...	624	53	36,88	0,03629
GO:0001947	heart looping	89	10	5,26	0,03682
GO:0071773	cellular response to BMP stimulus	170	19	10,05	0,03684
GO:0045596	negative regulation of cell differentiat...	679	71	40,13	0,03698
GO:0014033	neural crest cell differentiation	100	13	5,91	0,03708
GO:0048705	skeletal system morphogenesis	269	29	15,9	0,03734
GO:0031017	exocrine pancreas development	22	4	1,3	0,03782
GO:0072079	nephron tubule formation	22	4	1,3	0,03782
GO:0072215	regulation of metanephros development	22	4	1,3	0,03782
GO:0060259	regulation of feeding behavior	32	5	1,89	0,03805
GO:2000036	regulation of stem cell population maint...	32	5	1,89	0,03805
GO:0001662	behavioral fear response	54	7	3,19	0,03869

GO:0045471	response to ethanol	208	19	12,29	0,03921
GO:0060612	adipose tissue development	43	6	2,54	0,03943
GO:0007223	Wnt signaling pathway, calcium modulatin...	43	6	2,54	0,03943
GO:0007218	neuropeptide signaling pathway	78	9	4,61	0,03998
GO:0034220	monoatomic ion transmembrane transport	1117	57	66,02	0,04054
GO:0031323	regulation of cellular metabolic process	5298	350	313,16	0,04139
GO:0070301	cellular response to hydrogen peroxide	91	10	5,38	0,04194
GO:0071236	cellular response to antibiotic	169	16	9,99	0,04211
GO:0006935	chemotaxis	532	37	31,45	0,0422
GO:0001892	embryonic placenta development	93	15	5,5	0,04249
GO:0042475	odontogenesis of dentin-containing tooth	85	11	5,02	0,04253
GO:0046677	response to antibiotic	421	39	24,88	0,04256
GO:0048596	embryonic camera-type eye morphogenesis	45	7	2,66	0,04341
GO:0007520	myoblast fusion	40	6	2,36	0,04354
GO:0060602	branch elongation of an epithelium	23	4	1,36	0,04372
GO:0071498	cellular response to fluid shear stress	23	4	1,36	0,04372
GO:0031641	regulation of myelination	41	5	2,42	0,04373
GO:0001501	skeletal system development	556	52	32,86	0,04395
GO:0060008	Sertoli cell differentiation	32	7	1,89	0,04426
GO:0002068	glandular epithelial cell development	33	5	1,95	0,04449
GO:0031102	neuron projection regeneration	70	7	4,14	0,04451
GO:0009200	deoxyribonucleoside triphosphate metabol...	23	4	1,36	0,04453
GO:0009746	response to hexose	262	18	15,49	0,04454
GO:0048644	muscle organ morphogenesis	78	7	4,61	0,04456
GO:0060571	morphogenesis of an epithelial fold	24	3	1,42	0,04465
GO:0050680	negative regulation of epithelial cell p...	118	11	6,97	0,04596
GO:0060997	dendritic spine morphogenesis	63	5	3,72	0,04606

GO:0042476	odontogenesis	128	17	7,57	0,04632
GO:0008219	cell death	1862	128	110,06	0,04651
GO:0014031	mesenchymal cell development	93	10	5,5	0,04754
GO:0017085	response to insecticide	34	5	2,01	0,04778
GO:0021772	olfactory bulb development	34	5	2,01	0,04778
GO:0021854	hypothalamus development	34	5	2,01	0,04778
GO:0048703	embryonic viscerocranium morphogenesis	34	5	2,01	0,04778
GO:0035116	embryonic hindlimb morphogenesis	34	5	2,01	0,04778
GO:0043408	regulation of MAPK cascade	684	58	40,43	0,04821
GO:0007189	adenylate cyclase-activating G protein-c...	94	13	5,56	0,04878
GO:0071300	cellular response to retinoic acid	81	9	4,79	0,04903
GO:0014075	response to amine	70	9	4,14	0,04963
GO:0035176	social behavior	57	7	3,37	0,04971
GO:0051865	protein autoubiquitination	57	7	3,37	0,04971

Supplementary Table 10

Gene ID	Description	Gene name
HarAnt_Hap1.g165	B-cell CLL lymphoma 6a (zinc finger protein 51)	BCL6
HarAnt_Hap1.g201	Tubulin is the major constituent of microtubules. It binds two moles of GTP, one at an exchangeable site on the beta chain and one at a non-exchangeable site on the alpha chain	TUBB4A
HarAnt_Hap1.g348	Prostaglandin E synthase	PTGES2
HarAnt_Hap1.g509	acetoacetyl-CoA synthetase	AACS
HarAnt_Hap1.g742	Bone morphogenetic protein receptor	BMPR1B
HarAnt_Hap1.g11031	7-dehydrocholesterol reductase	DHCR7
HarAnt_Hap1.g11170	ERO1-like (S. cerevisiae)	ERO1L
HarAnt_Hap1.g11253	Belongs to the TRAFAC class myosin-kinesin ATPase superfamily. Kinesin family	KIF23
HarAnt_Hap1.g11414	acyl-CoA synthetase family member 3	ACSF3
HarAnt_Hap1.g15453	Phospholipase C eta	PLCH2
HarAnt_Hap1.g15533	Itchy E3 ubiquitin protein ligase	ITCH
HarAnt_Hap1.g15598	Nephronophthisis 4	NPHP4
HarAnt_Hap1.g15610	Oxysterol binding protein-like 2b	OSBPL2
HarAnt_Hap1.g16923	MICAL-like	MICALL2
HarAnt_Hap1.g16994	Centlein, centrosomal protein	CNTLN
HarAnt_Hap1.g17019	Type II inositol 3,4-bisphosphate	INPP4B
HarAnt_Hap1.g17230	regulatory subunit	PIK3R6
HarAnt_Hap1.g17414	3-hydroxybutyrate dehydrogenase, type 2	BDH2
HarAnt_Hap1.g18372	Solute carrier family 9, subfamily A (NHE1, cation proton antiporter 1), member 1	SLC9A1
HarAnt_Hap1.g18497	ubiquitin-conjugating enzyme	UBE2E1
HarAnt_Hap1.g19011	Zinc binding alcohol dehydrogenase domain containing 2	ZADH2
HarAnt_Hap1.g19098	Abhydrolase domain containing	ABHD5
HarAnt_Hap1.g19316	Breast carcinoma amplified sequence 3	BCAS3
HarAnt_Hap1.g19327	Aldehyde dehydrogenase 3 family, member A2a	ALDH3A2
HarAnt_Hap1.g19581	Schwannomin interacting protein 1	SCHIP1
HarAnt_Hap1.g19951	Dynein assembly factor with WDR repeat domains	DAW1
HarAnt_Hap1.g20305	Peroxisome biogenesis factor 13	PEX13

HarAnt_Hap1.g20461	Endoplasmic reticulum lectin 1	ERLEC1
HarAnt_Hap1.g20659	Pleckstrin	PLEK
HarAnt_Hap1.g20790	Serologically defined colon cancer antigen 8	SDCCAG8
HarAnt_Hap1.g20796	F-box protein 5	FBXO5
HarAnt_Hap1.g20797	Anaphase promoting complex subunit 1	ANAPC1
HarAnt_Hap1.g20853	LIM-domain binding factor 3a	LDB3
HarAnt_Hap1.g21269	Microtubule associated monooxygenase, calponin and LIM domain containing	MICAL3
HarAnt_Hap1.g21272	Thromboxane A synthase 1 (platelet, cytochrome P450, family 5, subfamily A)	TBXAS1
HarAnt_Hap1.g21368	v-Ki-ras2 Kirsten rat sarcoma viral oncogene homolog	KRAS
HarAnt_Hap1.g21495	Nuclear receptor subfamily 2, group F, member 2	NR2F2
HarAnt_Hap1.g21756	caldesmon 1	CALD1
HarAnt_Hap1.g21837	Cellular retinoic acid binding protein	CRABP1
HarAnt_Hap1.g22833	Galactosidase, alpha	GLA
HarAnt_Hap1.g22945	Sprouty homolog	SPRY1
HarAnt_Hap1.g22963	coiled-coil	CCDC69
HarAnt_Hap1.g1941	pre-B-cell leukemia transcription factor	PBX1
HarAnt_Hap1.g2173	Kelch-like 20 (Drosophila)	KLHL20
HarAnt_Hap1.g2195	Fatty acid binding protein 6, ileal (gastrotropin)	FABP6
HarAnt_Hap1.g2369	Belongs to the profilin family	PFN2
HarAnt_Hap1.g2503	homolog subfamily B member	DNAJB6
HarAnt_Hap1.g2516	neuronal	ANK2
HarAnt_Hap1.g2640	Insulin-induced gene	INSIG2
HarAnt_Hap1.g2811	Belongs to the TRAFAC class myosin-kinesin ATPase superfamily. Myosin family	MYO1B
HarAnt_Hap1.g2877	erythroblastic leukemia viral oncogene homolog	ERBB4
HarAnt_Hap1.g2997	Insulin receptor substrate	IRS2
HarAnt_Hap1.g3225	Nuclear receptor subfamily 0 group B member	NR0B1
HarAnt_Hap1.g3229	Tripartite motif-containing 13	TRIM13
HarAnt_Hap1.g3557	casein kinase	CSNK2A1
HarAnt_Hap1.g3562	Myotubularin related protein 14	MTMR14
HarAnt_Hap1.g3668	Zinc finger protein	ZPR1
HarAnt_Hap1.g3674	Dehydrogenase reductase SDR family member	DHRS3
HarAnt_Hap1.g3741	Solute carrier family 16 (monocarboxylate transporter), member 1	SLC16A1

HarAnt_Hap1.g4675	Hydroxyacid oxidase (glycolate oxidase) 1	HAO1
HarAnt_Hap1.g4753	intermediate filament bundle assembly	KRT14
HarAnt_Hap1.g4773	Family with sequence similarity 57, member	FAM57B
HarAnt_Hap1.g4776	CDP-diacylglycerol--inositol 3-phosphatidyltransferase	CDIPT
HarAnt_Hap1.g4782	forkhead box	FOXJ1
HarAnt_Hap1.g4808	Ectonucleotide pyrophosphatase phosphodiesterase	ENPP7
HarAnt_Hap1.g5000	Speckle-type POZ	SPOP
HarAnt_Hap1.g5225	SRY (sex determining region Y)-box	SOX9
HarAnt_Hap1.g5502	Sphingosine kinase	SPHK1
HarAnt_Hap1.g6062	Thioredoxin-related transmembrane protein	TMX1
HarAnt_Hap1.g6063	FERM domain containing 6	FRMD6
HarAnt_Hap1.g6064	Guanine nucleotide-binding proteins (G proteins) are involved as a modulator or transducer in various transmembrane signaling systems. The beta and gamma chains are required for the GTPase activity, for replacement of GDP by GTP, and for G protein- effector interaction	GNG2
HarAnt_Hap1.g6123	Lipid phosphate phosphatase-related protein type	PLPPR3
HarAnt_Hap1.g6191	AHA1, activator of heat shock protein ATPase homolog 1, like	AHSA1
HarAnt_Hap1.g6207	Zinc finger, FYVE	ZFYVE21
HarAnt_Hap1.g6209	BTB POZ domain-containing protein	BTBD6
HarAnt_Hap1.g6608	protein domain specific binding	IPCEF1
HarAnt_Hap1.g6664	activator of morphogenesis	DAAM2
HarAnt_Hap1.g6693	Rho-associated, coiled-coil containing protein kinase 2a	ROCK2
HarAnt_Hap1.g6711	Tubulin is the major constituent of microtubules. It binds two moles of GTP, one at an exchangeable site on the beta chain and one at a non-exchangeable site on the alpha chain	TUBB4A
HarAnt_Hap1.g6811	Responsible for the deiodination of T4 (3,5,3',5'- tetraiodothyronine)	DIO2
HarAnt_Hap1.g7216	acyl-Coenzyme A binding domain containing 3	ACBD3
HarAnt_Hap1.g7832	complement	C3
HarAnt_Hap1.g7835	Serine incorporator	SERINC2
HarAnt_Hap1.g7896	Abhydrolase domain containing	ABHD5
HarAnt_Hap1.g7970	HEPACAM family member 2	HEPACAM2
HarAnt_Hap1.g8477	Metastasis suppressor	MTSS1
HarAnt_Hap1.g8736	Anaphase-promoting complex subunit 4 WD40 domain	FZR1
HarAnt_Hap1.g9020	Mast stem cell growth factor receptor	KIT
HarAnt_Hap1.g9052	Guanine nucleotide binding protein (G protein), beta polypeptide	GNB4

HarAnt_Hap1.g9226	synthase	PTGS2
HarAnt_Hap1.g9280	Cell surface proteoglycan that bears heparan sulfate	GPC1
HarAnt_Hap1.g9282	large homolog	dlg1
HarAnt_Hap1.g9392	regulatory subunit	PIK3R3
HarAnt_Hap1.g9792	P21 protein (Cdc42 Rac)-activated kinase 3	PAK3
HarAnt_Hap1.g9997	Dihydropyrimidinase-like 3	DPYSL3
HarAnt_Hap1.g10065	Bromodomain and WD	BRWD3
HarAnt_Hap1.g10172	Active breakpoint cluster region-related	ABR
HarAnt_Hap1.g10195	Peroxiredoxin 5	PRDX5
HarAnt_Hap1.g10596	Sulfotransferase family 4A, member 1	SULT4A1
HarAnt_Hap1.g10628	bicaudal D homolog	BICD1
HarAnt_Hap1.g10639	phytanoyl-CoA	PHYH
HarAnt_Hap1.g10653	Phosphoinositide-3-kinase, catalytic, gamma polypeptide	PIK3CG
HarAnt_Hap1.g10723	tyrosine	TH
HarAnt_Hap1.g10850	ubiquitin-conjugating enzyme	UBE2H
HarAnt_Hap1.g11986	Dolichyl pyrophosphate phosphatase 1	DOLPP1
HarAnt_Hap1.g12036	Tubulin tyrosine ligase-like family, member 11	TTLL11
HarAnt_Hap1.g12057	Phosphoinositide-3-kinase, regulatory subunit 1 (p85 alpha)	PIK3R1
HarAnt_Hap1.g12451	Lipid phosphate phosphatase-related protein type	PLPPR1
HarAnt_Hap1.g12474	Phosphatidylinositol glycan anchor biosynthesis, class O	PIGO
HarAnt_Hap1.g12483	Retinoid X receptor, alpha a	RXRA
HarAnt_Hap1.g13084	Zinc finger, RAN-binding domain containing	ZRANB1
HarAnt_Hap1.g13109	F-box and leucine-rich repeat protein 15	FBXL15
HarAnt_Hap1.g13112	Belongs to the fatty acid desaturase type 1 family	SCD
HarAnt_Hap1.g14033	Signal sequence receptor, alpha	SSR1
HarAnt_Hap1.g14034	Nebulette	NEBL
HarAnt_Hap1.g14041	TRAF2 and NCK interacting kinase	TNIK
HarAnt_Hap1.g14147	Spindle and centriole associated protein 1	SPICE1
HarAnt_Hap1.g14395	ADP-ribosylation factor-like	ARL2
HarAnt_Hap1.g14446	Belongs to the glycosyl hydrolase 1 family	GBA3
HarAnt_Hap1.g14454	Tyrosinase-related protein	TYRP1

Supplementary Table 11

Gene	OG	pvalue	fdr
HarAnt_Hap1.g10130	OG0009506	0.0107	0.234886438356164
HarAnt_Hap1.g10161	OG0009494	0.00443	0.145734863013699
HarAnt_Hap1.g10253	OG0001634	0.00119	0.0642198876404494
HarAnt_Hap1.g10484	OG0011405	0.01526	0.278683574144487
HarAnt_Hap1.g10561	OG0011384	0.00027	0.0275917021276596
HarAnt_Hap1.g10589	OG0011377	0.01401	0.27300252
HarAnt_Hap1.g10642	OG0011356	0.00046	0.036823
HarAnt_Hap1.g10676	OG0011344	0.03273	0.388153555555556
HarAnt_Hap1.g10772	OG0011312	0.00265	0.102644758064516
HarAnt_Hap1.g1087	OG0001673	0.00229	0.0981125641025641
HarAnt_Hap1.g1105	OG0011240	0.02097	0.329146764705882
HarAnt_Hap1.g11110	OG0000739	0.00545	0.169336538461538
HarAnt_Hap1.g11265	OG0005564	0.02292	0.338567300613497
HarAnt_Hap1.g11298	OG0008769	0.00484	0.153950463576159
HarAnt_Hap1.g11318	OG0008777	0.02524	0.345651794871795
HarAnt_Hap1.g11319	OG0008778	0.03542	0.400287670588235
HarAnt_Hap1.g11348	OG0005572	0.01792	0.302572264808362
HarAnt_Hap1.g11356	OG0008796	0.02651	0.354570498614958
HarAnt_Hap1.g11486	OG0001575	6e-05	0.009606
HarAnt_Hap1.g11501	OG0008845	0.02178	0.336364437299035
HarAnt_Hap1.g11654	OG0000841	2e-05	0.00505578947368421
HarAnt_Hap1.g11700	OG0000974.ab	0.02453	0.345651794871795
HarAnt_Hap1.g12026	OG0009182	0.01002	0.228085592417062
HarAnt_Hap1.g12093	OG0009202	0.01428	0.273254342629482
HarAnt_Hap1.g12096	OG0009203	0.00793	0.211598833333333
HarAnt_Hap1.g12118	OG0009215	0.0423	0.440709110629067
HarAnt_Hap1.g12122	OG0009219	0.03604	0.403496783216783
HarAnt_Hap1.g12164	OG0009226	0.0199	0.320737248322148
HarAnt_Hap1.g12166	OG0009228	0.0466	0.456723407707911
HarAnt_Hap1.g12234	OG0009245	0.01652	0.289227927272727

HarAnt_Hap1.g12333	OG0009282	0.04533	0.449169444444444
HarAnt_Hap1.g12339	OG0009286	0.01153	0.245250530973451
HarAnt_Hap1.g12352	OG0005736	0.00029	0.029018125
HarAnt_Hap1.g12360	OG0009294	0.00255	0.0995743902439024
HarAnt_Hap1.g12399	OG0009308	0.03381	0.397040171149144
HarAnt_Hap1.g12527	OG0009345	0.03783	0.415053767123288
HarAnt_Hap1.g12630	OG0005682	0.00229	0.0981125641025641
HarAnt_Hap1.g12855	OG0006645	0.00576	0.172908
HarAnt_Hap1.g12870	OG0009887	0.00075	0.05003125
HarAnt_Hap1.g12901	OG0009894	0.009	0.220512118226601
HarAnt_Hap1.g12957	OG0001192.aa	0.03723	0.412017718894009
HarAnt_Hap1.g13023	OG0001016	0.01808	0.302572264808362
HarAnt_Hap1.g13104	OG0009947	0.00406	0.13829914893617
HarAnt_Hap1.g13125	OG0009955	0.04227	0.440709110629067
HarAnt_Hap1.g13167	OG0000957.ab	0.04316	0.442773375796178
HarAnt_Hap1.g13203	OG0009977	0.00255	0.0995743902439024
HarAnt_Hap1.g13247	OG0009988	0.04674	0.456723407707911
HarAnt_Hap1.g13277	OG0009997	2e-05	0.00505578947368421
HarAnt_Hap1.g13297	OG0010000	1e-05	0.00369461538461538
HarAnt_Hap1.g13392	OG0010023	0.00236	0.0981125641025641
HarAnt_Hap1.g13489	OG0004013	0.01141	0.2446528125
HarAnt_Hap1.g13680	OG0003655	0.00041	0.0364672222222222
HarAnt_Hap1.g13707	OG0003870	0.00864	0.217266596858639
HarAnt_Hap1.g13777	OG0010868	0.0055	0.169336538461538
HarAnt_Hap1.g13858	OG0010891	0.04875	0.471215855130785
HarAnt_Hap1.g13928	OG0010905	0.00048	0.0371845161290323
HarAnt_Hap1.g13973	OG0010915	0.0409	0.431742197802198
HarAnt_Hap1.g14024	OG0010934	0.00055	0.040025
HarAnt_Hap1.g14097	OG0004560	0.00994	0.228085592417062
HarAnt_Hap1.g14144	OG0010946	0.03014	0.374089329896907
HarAnt_Hap1.g14514	OG0006595	0.00192	0.0899979611650486
HarAnt_Hap1.g15118	OG0009418	0.03022	0.374089329896907
HarAnt_Hap1.g15177	OG0009408	0.01001	0.228085592417062

HarAnt_Hap1.g15256	OG0009388	0.00504	0.15884431372549
HarAnt_Hap1.g15337	OG0009373	0.03109	0.376134181360201
HarAnt_Hap1.g15479	OG0007257	0.02647	0.354570498614958
HarAnt_Hap1.g15539	OG0007269	0.03666	0.409483674418605
HarAnt_Hap1.g15553	OG0007274	0.03201	0.381498833746898
HarAnt_Hap1.g15806	OG0007362	0.03899	0.421285560538117
HarAnt_Hap1.g15832	OG0007368	0.02378	0.342681107784431
HarAnt_Hap1.g15868	OG0007378	0.03963	0.424872522321429
HarAnt_Hap1.g15885	OG0007384	5e-04	0.0375234375
HarAnt_Hap1.g15895	OG0001258	0.01154	0.245250530973451
HarAnt_Hap1.g15939	OG0007400	0.01976	0.320632702702703
HarAnt_Hap1.g16000	OG0002070	0.03765	0.414754472477064
HarAnt_Hap1.g16005	OG0001513	0.00338	0.118497372262774
HarAnt_Hap1.g16018	OG0001514	0.015	0.278683574144487
HarAnt_Hap1.g16105	OG0007453	0	0
HarAnt_Hap1.g16218	OG0007482	0.00876	0.218001450777202
HarAnt_Hap1.g16227	OG0007487	0.00108	0.0596234482758621
HarAnt_Hap1.g16249	OG0001517	0.00921	0.220512118226601
HarAnt_Hap1.g16941	OG0011623	0.0149	0.278683574144487
HarAnt_Hap1.g17033	OG0011595	0.02168	0.335900129032258
HarAnt_Hap1.g17113	OG0011568	0.01525	0.278683574144487
HarAnt_Hap1.g17157	OG0000437.aa	0.03909	0.421285560538117
HarAnt_Hap1.g17222	OG0000907	0.00506	0.15884431372549
HarAnt_Hap1.g17479	OG0011475	0.04962	0.471757114624506
HarAnt_Hap1.g17616	OG0011439	0.00835	0.214618263157895
HarAnt_Hap1.g17670	OG0011423	0.01749	0.300015964285714
HarAnt_Hap1.g17742	OG0000919	0.03072	0.376134181360201
HarAnt_Hap1.g17757	OG0006380	0.00882	0.218363195876289
HarAnt_Hap1.g1798	OG0000390	0.01557	0.279039962686567
HarAnt_Hap1.g18355	OG0007736	0.00023	0.0251065909090909
HarAnt_Hap1.g18358	OG0007735	0.00055	0.040025
HarAnt_Hap1.g18490	OG0007707	0.02796	0.362951027027027
HarAnt_Hap1.g18560	OG0001528	6e-05	0.009606
HarAnt_Hap1.g18610	OG0005074	0.00295	0.110694140625

HarAnt_Hap1.g1886	OG0008410	0.01232	0.259530526315789
HarAnt_Hap1.g19155	OG0006631	0.01515	0.278683574144487
HarAnt_Hap1.g19400	OG0009705	0.00842	0.214618263157895
HarAnt_Hap1.g19415	OG0009713	0.00125	0.0667083333333333
HarAnt_Hap1.g19421	OG0009716	0.02882	0.367168328912467
HarAnt_Hap1.g19470	OG0009729	0.00316	0.114116390977444
HarAnt_Hap1.g19591	OG0009764	0.00103	0.0587661176470588
HarAnt_Hap1.g19600	OG0009766	0.00614	0.18203962962963
HarAnt_Hap1.g19618	OG0009776	0.01055	0.234590972222222
HarAnt_Hap1.g19663	OG0009794	0.03153	0.379808304239402
HarAnt_Hap1.g1967	OG0008390	0.04917	0.471757114624506
HarAnt_Hap1.g1969	OG0001972	0.00097	0.0568159756097561
HarAnt_Hap1.g19702	OG0009810	0.04708	0.457743400809717
HarAnt_Hap1.g19794	OG0009844	0.02261	0.338567300613497
HarAnt_Hap1.g19838	OG0009852	0.03579	0.403496783216783
HarAnt_Hap1.g19908	OG0009870	0.0105	0.23456511627907
HarAnt_Hap1.g19938	OG0009876	0.01136	0.2446528125
HarAnt_Hap1.g1994	OG0008382	0.0027	0.1037448
HarAnt_Hap1.g20245	OG0010486	0.00335	0.118309191176471
HarAnt_Hap1.g20258	OG0010491	0.04688	0.456723407707911
HarAnt_Hap1.g20324	OG0010509	0.04106	0.432480657894737
HarAnt_Hap1.g20348	OG0010518	0.01065	0.234886438356164
HarAnt_Hap1.g20517	OG0010564	0.01583	0.281598111111111
HarAnt_Hap1.g2058	OG0008362	0.00664	0.189832857142857
HarAnt_Hap1.g20587	OG0003322	0.03197	0.381498833746898
HarAnt_Hap1.g20608	OG0006134	0.00046	0.036823
HarAnt_Hap1.g20620	OG0010596	0.00044	0.036823
HarAnt_Hap1.g20633	OG0010603	0.02351	0.342681107784431
HarAnt_Hap1.g2064	OG0008361	0.03977	0.425423853006682
HarAnt_Hap1.g20678	OG0010615	0.00924	0.220512118226601
HarAnt_Hap1.g2112	OG0008348	0.00574	0.172908
HarAnt_Hap1.g21309	OG0005288	0.04966	0.471757114624506
HarAnt_Hap1.g21320	OG0005293	0.01071	0.234886438356164
HarAnt_Hap1.g21379	OG0000155.ab	0.0083	0.214618263157895

HarAnt_Hap1.g21388	OG0005313	0.00679	0.190715614035088
HarAnt_Hap1.g21463	OG0000973.aa	0.02695	0.355606730769231
HarAnt_Hap1.g21482	OG0005338	0.03104	0.376134181360201
HarAnt_Hap1.g21564	OG0005357	0.02277	0.338567300613497
HarAnt_Hap1.g21638	OG0008180	0.01384	0.27300252
HarAnt_Hap1.g21659	OG0005365	0.04687	0.456723407707911
HarAnt_Hap1.g21847	OG0010461	2e-05	0.00505578947368421
HarAnt_Hap1.g21863	OG0000980.aa	0.02358	0.342681107784431
HarAnt_Hap1.g21904	OG0000825.aa	0.01674	0.291312391304348
HarAnt_Hap1.g21926	OG0003216	0.00013	0.0173441666666667
HarAnt_Hap1.g22086	OG0004215	0.00239	0.0981125641025641
HarAnt_Hap1.g22218	OG0006919	0.0369	0.41025625
HarAnt_Hap1.g22280	OG0010043	0.03785	0.415053767123288
HarAnt_Hap1.g22358	OG0002120	0.02767	0.360159918699187
HarAnt_Hap1.g22416	OG0010091	0.02269	0.338567300613497
HarAnt_Hap1.g22424	OG0010094	0.02256	0.338567300613497
HarAnt_Hap1.g22493	OG0000157	0	0
HarAnt_Hap1.g22552	OG0010142	0.00698	0.193785780346821
HarAnt_Hap1.g22785	OG0010201	0.00977	0.227792766990291
HarAnt_Hap1.g22822	OG0010215	0.00316	0.114116390977444
HarAnt_Hap1.g22905	OG0010240	0.03046	0.375988692307692
HarAnt_Hap1.g2504	OG0005406	0.03011	0.374089329896907
HarAnt_Hap1.g2836	OG0004222	0.03535	0.400287670588235
HarAnt_Hap1.g290	OG0010984	0.0045	0.146037162162162
HarAnt_Hap1.g2924	OG0008586	9e-05	0.0127138235294118
HarAnt_Hap1.g2956	OG0008595	0.00079	0.0505578947368421
HarAnt_Hap1.g2965	OG0008597	0.0083	0.214618263157895
HarAnt_Hap1.g3003	OG0008608	0.01303	0.269754698275862
HarAnt_Hap1.g3175	OG0008661	0.00021	0.024015
HarAnt_Hap1.g3193	OG0008667	0.04545	0.449169444444444
HarAnt_Hap1.g3238	OG0008681	0.02874	0.367168328912467
HarAnt_Hap1.g3271	OG0008691	0.00899	0.220512118226601

HarAnt_Hap1.g3311	OG0008695	0.03737	0.412616344827586
HarAnt_Hap1.g3401	OG0004113	0.02111	0.329504512987013
HarAnt_Hap1.g3605	OG0004870	3e-05	0.0057636
HarAnt_Hap1.g3743	OG0006967	0.04598	0.452208834355828
HarAnt_Hap1.g3777	OG0006981	3e-05	0.0057636
HarAnt_Hap1.g390	OG0011010	0.00251	0.0995743902439024
HarAnt_Hap1.g3921	OG0003447	0.00422	0.142737042253521
HarAnt_Hap1.g4198	OG0007130	0.03483	0.400287670588235
HarAnt_Hap1.g4209	OG0000429.ab	0.00383	0.131396357142857
HarAnt_Hap1.g4214	OG0004908	0.01421	0.27300252
HarAnt_Hap1.g4276	OG0007150	0.00983	0.228084492753623
HarAnt_Hap1.g4310	OG0007159	0.02748	0.359636076294278
HarAnt_Hap1.g4447	OG0007211	0.02906	0.368272242744063
HarAnt_Hap1.g4509	OG0001278	0.01782	0.302436254416961
HarAnt_Hap1.g456	OG0001405	0.00206	0.0924689719626168
HarAnt_Hap1.g4697	OG0007793	0.04007	0.427680466666667
HarAnt_Hap1.g4721	OG0007799	8e-04	0.0505578947368421
HarAnt_Hap1.g486	OG0011039	0.02189	0.3366703514377
HarAnt_Hap1.g4956	OG0007844	0.02526	0.345651794871795
HarAnt_Hap1.g4958	OG0007846	0.01911	0.314333321917808
HarAnt_Hap1.g4972	OG0001098.aa	0.01046	0.23456511627907
HarAnt_Hap1.g4994	OG0007860	9e-05	0.0127138235294118
HarAnt_Hap1.g5034	OG0007874	0.03165	0.379808304239402
HarAnt_Hap1.g5096	OG0007883	0.01402	0.27300252
HarAnt_Hap1.g513	OG0011051	0.0226	0.338567300613497
HarAnt_Hap1.g5191	OG0007907	0.01095	0.239058409090909
HarAnt_Hap1.g525	OG0011053	0.0427	0.442414267241379
HarAnt_Hap1.g5321	OG0007939	0.0497	0.471757114624506
HarAnt_Hap1.g534	OG0004579	0.0346	0.400287670588235
HarAnt_Hap1.g5711	OG0003652	0.03171	0.379808304239402
HarAnt_Hap1.g576	OG0011064	0.02718	0.35765901369863
HarAnt_Hap1.g5811	OG0003172	0.02605	0.35285138028169
HarAnt_Hap1.g586	OG0011068	0.02009	0.3222813

HarAnt_Hap1.g5978	OG0003073	0.02578	0.351765170454545
HarAnt_Hap1.g6020	OG0003506	0.02502	0.345651794871795
HarAnt_Hap1.g604	OG0011076	0.04326	0.442773375796178
HarAnt_Hap1.g6154	OG0002341	0.03848	0.417199638826185
HarAnt_Hap1.g6225	OG0006855	0.04319	0.442773375796178
HarAnt_Hap1.g6257	OG0000786.aa	0.01544	0.279005730337079
HarAnt_Hap1.g6366	OG0006806	0.03299	0.388948823529412
HarAnt_Hap1.g6471	OG0006765	0.02951	0.370320078328982
HarAnt_Hap1.g65	OG0008961	0.00664	0.189832857142857
HarAnt_Hap1.g6518	OG0006747	0.02659	0.354570498614958
HarAnt_Hap1.g6525	OG0001486	0.02593	0.352809603399433
HarAnt_Hap1.g6609	OG0006725	0.02524	0.345651794871795
HarAnt_Hap1.g6659	OG0006709	0.03827	0.417199638826185
HarAnt_Hap1.g6774	OG0004779	0.00308	0.112925496183206
HarAnt_Hap1.g6970	OG0010690	0.01764	0.300442978723404
HarAnt_Hap1.g7015	OG0010704	0.00218	0.0969494444444444
HarAnt_Hap1.g7073	OG0010724	0.0141	0.27300252
HarAnt_Hap1.g7075	OG0010725	0.00816	0.214618263157895
HarAnt_Hap1.g7114	OG0010740	0.00193	0.0899979611650486
HarAnt_Hap1.g7158	OG0000457.ab	0.04028	0.427816887417219
HarAnt_Hap1.g7257	OG0010789	0.02291	0.338567300613497
HarAnt_Hap1.g7284	OG0010795	0.03518	0.400287670588235
HarAnt_Hap1.g7297	OG0004526	0.00238	0.0981125641025641
HarAnt_Hap1.g7421	OG0010832	0.00248	0.0995743902439024
HarAnt_Hap1.g7825	OG0000885.ab	0.01653	0.289227927272727
HarAnt_Hap1.g7866	OG0010449	0.00073	0.0493829577464789
HarAnt_Hap1.g7970	OG0010422	0.00161	0.0805503125
HarAnt_Hap1.g8061	OG0010388	0.0019	0.0899979611650486
HarAnt_Hap1.g8076	OG0010379	0.03542	0.400287670588235
HarAnt_Hap1.g8151	OG0001306	0.01863	0.309379448275862
HarAnt_Hap1.g8177	OG0006044	0.02274	0.338567300613497
HarAnt_Hap1.g8237	OG0010326	0.00457	0.1463314

HarAnt_Hap1.g830	OG0011145	0.02659	0.354570498614958
HarAnt_Hap1.g8373	OG0002562	0.04035	0.427816887417219
HarAnt_Hap1.g8514	OG0006022	0.0111	0.24015
HarAnt_Hap1.g859	OG0011159	5e-04	0.0375234375
HarAnt_Hap1.g8846	OG0009131	0.02013	0.3222813
HarAnt_Hap1.g893	OG0011174	0.01372	0.27300252
HarAnt_Hap1.g8947	OG0009096	0.03912	0.421285560538117
HarAnt_Hap1.g8971	OG0009089	4e-04	0.0362490566037736
HarAnt_Hap1.g8977	OG0009088	8e-05	0.0123948387096774
HarAnt_Hap1.g899	OG0011175	0.02415	0.343741331360947
HarAnt_Hap1.g9020	OG0009074	0.01302	0.269754698275862
HarAnt_Hap1.g9040	OG0009065	0.01551	0.279005730337079
HarAnt_Hap1.g9261	OG0009008	0.03166	0.379808304239402
HarAnt_Hap1.g9426	OG0004281	0.00738	0.201398522727273
HarAnt_Hap1.g943	OG0006318	0.00588	0.175413913043478
HarAnt_Hap1.g946	OG0011186	0.00849	0.214618263157895
HarAnt_Hap1.g962	OG0005765	0.00736	0.201398522727273
HarAnt_Hap1.g9633	OG0009651	0.00108	0.0596234482758621
HarAnt_Hap1.g964	OG0011192	0.02924	0.368608188976378
HarAnt_Hap1.g9672	OG0009639	0.01549	0.279005730337079
HarAnt_Hap1.g9706	OG0009629	0.00046	0.036823
HarAnt_Hap1.g9804	OG0000437.ab	3e-05	0.0057636
HarAnt_Hap1.g9811	OG0009588	0.01392	0.27300252
HarAnt_Hap1.g9929	OG0009564	0.04961	0.471757114624506
HarAnt_Hap1.g9952	OG0005838	0.01619	0.286939372693727
HarAnt_Hap1.g996	OG0011206	0.02476	0.345651794871795
HarAnt_Hap1.g997	OG0011207	2e-04	0.0234292682926829
HarAnt_Hap1.g9985	OG0009549	0.00785	0.210634357541899

Supplementary Table 12

GO.ID	Term	Annotated	Significant	Expected	classicFisher
**GO:0043524**	negative regulation of neuron apoptotic process	55	12	3,83	0,00029
**GO:0010501**	RNA secondary structure unwinding	13	5	0,9	0,00127
**GO:0030595**	leukocyte chemotaxis	28	4	1,95	0,00302
**GO:0003407**	neural retina development	33	7	2,3	0,00307
**GO:0071353**	cellular response to interleukin-4	10	4	0,7	0,00343
**GO:0014031**	mesenchymal cell development	23	6	1,6	0,00393
**GO:0071902**	positive regulation of protein serine/threonine kinase activity	81	11	5,64	0,00614
**GO:0035886**	vascular associated smooth muscle cell differentiation	12	4	0,83	0,00724
**GO:0014032**	neural crest cell development	23	5	1,6	0,00934
**GO:0055123**	digestive system development	58	4	4,04	0,00972
**GO:0032092**	positive regulation of protein binding	28	6	1,95	0,01094
**GO:0006690**	icosanoid metabolic process	22	4	1,53	0,01372
**GO:0046031**	ADP metabolic process	28	3	1,95	0,01388
**GO:1903313**	positive regulation of mRNA metabolic process	30	3	2,09	0,0139
**GO:0031503**	protein-containing complex localization	47	8	3,27	0,01434
**GO:0019221**	cytokine-mediated signaling pathway	126	14	8,77	0,01545
**GO:0007030**	Golgi organization	39	7	2,71	0,01629
**GO:0032496**	response to lipopolysaccharide	80	11	5,57	0,02058
**GO:0007596**	blood coagulation	73	9	5,08	0,02078
**GO:0045665**	negative regulation of neuron differentiation	61	9	4,24	0,02352
**GO:0046425**	regulation of receptor signaling pathway via JAK-STAT	30	6	2,09	0,02588
**GO:0006220**	pyrimidine nucleotide metabolic process	16	4	1,11	0,02609
**GO:0042310**	vasoconstriction	21	4	1,46	0,02616
**GO:0006361**	transcription initiation at RNA polymerase I promoter	17	4	1,18	0,02646
**GO:0030521**	androgen receptor signaling pathway	17	4	1,18	0,02646
**GO:0051603**	obsolete proteolysis involved in protein catabolic process	174	16	12,11	0,02717
**GO:1901984**	negative regulation of protein acetylation	10	3	0,7	0,02768
**GO:0001910**	regulation of leukocyte mediated cytotoxicity	10	3	0,7	0,02768
**GO:0009303**	rRNA transcription	10	3	0,7	0,02768
**GO:1903727**	positive regulation of phospholipid metabolic process	10	3	0,7	0,02768
**GO:0051260**	protein homooligomerization	107	13	7,44	0,03246
**GO:0006084**	acetyl-CoA metabolic process	11	3	0,77	0,03615
**GO:0032481**	positive regulation of type I interferon production	11	3	0,77	0,03615

**GO:0047496**	vesicle transport along microtubule	11	3	0,77	0,03615
**GO:0000027**	ribosomal large subunit assembly	11	3	0,77	0,03615
**GO:0010039**	response to iron ion	11	3	0,77	0,03615
**GO:0000460**	maturation of 5.8S rRNA	11	3	0,77	0,03615
**GO:0045601**	regulation of endothelial cell differentiation	11	3	0,77	0,03615
**GO:0045089**	positive regulation of innate immune response	62	4	4,31	0,03715
**GO:0006364**	rRNA processing	63	10	4,38	0,03854
**GO:0045840**	positive regulation of mitotic nuclear division	19	4	1,32	0,03864
**GO:0043065**	positive regulation of apoptotic process	160	15	11,13	0,03867
**GO:0048732**	gland development	153	13	10,64	0,03879
**GO:0050768**	negative regulation of neurogenesis	78	11	5,43	0,04011
**GO:0070301**	cellular response to hydrogen peroxide	28	5	1,95	0,04111
**GO:1903047**	mitotic cell cycle process	204	16	14,19	0,04184
**GO:1902680**	positive regulation of RNA biosynthetic process	395	36	27,48	0,04262
**GO:0010605**	negative regulation of macromolecule metabolic process	620	42	43,14	0,04297
**GO:0006509**	membrane protein ectodomain proteolysis	12	3	0,83	0,04578
**GO:0051150**	regulation of smooth muscle cell differentiation	12	3	0,83	0,04578
**GO:0045824**	negative regulation of innate immune response	12	3	0,83	0,04578
**GO:0046427**	positive regulation of receptor signaling pathway via JAK-STAT	12	3	0,83	0,04578
**GO:0002474**	antigen processing and presentation of peptide antigen via MHC class I	12	3	0,83	0,04578
**GO:0008217**	regulation of blood pressure	45	5	3,13	0,04592

Supplementary Table 13

Hap1			
**Chr**	**Gene name**	**Status**	**Gene ID**
SUPER_5	tryp1	complete	Hap1.G00000000001
SUPER_5	tryp1	complete	Hap1.G00000000002
SUPER_5	tryp1	complete	Hap1.G00000000003
SUPER_5	tryp1	complete	Hap1.G00000000004
SUPER_5	tryp1	complete	Hap1.G00000000005
SUPER_5	tryp1	complete	Hap1.G00000000006
SUPER_5	tryp1	partial	Hap1.G00000000007
SUPER_5	tryp1	complete	Hap1.G00000000008
SUPER_5	tryp1	partial	Hap1.G00000000009
SUPER_5	tryp1	complete	Hap1.G00000000010
SUPER_5	tryp3	complete	Hap1.G00000000011
SUPER_5	tlp	complete	Hap1.G00000000012
SUPER_5	afgp1	partial	Hap1.G00000000013
SUPER_5	afgp2	complete	Hap1.G00000000014
SUPER_5	tryp3	partial	Hap1.G00000000015
SUPER_5	tryp3	partial	Hap1.G00000000016
SUPER_5	afgp3	complete	Hap1.G00000000017
SUPER_5	afgp4	partial	Hap1.G00000000018
SUPER_5	tryp3	partial	Hap1.G00000000019
SUPER_5	tryp3	partial	Hap1.G00000000020
SUPER_5	afgp5	complete	Hap1.G00000000021

SUPER_5	afgp6	complete	Hap1.G00000000022
SUPER_5	tryp3	partial	Hap1.G00000000023
SUPER_5	tryp3	partial	Hap1.G00000000024
SUPER_5	afgp7	complete	Hap1.G00000000025
SUPER_5	afgp8	complete	Hap1.G00000000026
SUPER_5	tryp3	partial	Hap1.G00000000027
SUPER_5	tryp3	partial	Hap1.G00000000028
SUPER_5	afgp9	complete	Hap1.G00000000029
SUPER_5	afgp10	complete	Hap1.G00000000030
SUPER_5	tryp3	partial	Hap1.G00000000031
SUPER_5	tryp3	partial	Hap1.G00000000032
SUPER_5	afgp11	complete	Hap1.G00000000033
SUPER_5	afgp12	complete	Hap1.G00000000034
SUPER_5	tryp3	partial	Hap1.G00000000035
SUPER_5	tryp3	partial	Hap1.G00000000036
SUPER_5	afgp13	complete	Hap1.G00000000037
SUPER_5	afgp14	complete	Hap1.G00000000038
SUPER_5	tryp3	partial	Hap1.G00000000039
SUPER_5	afgp15	complete	Hap1.G00000000040

SUPER_5	afgp/tlp1	complete	Hap1.G00000000041
SUPER_5	afgp16	complete	Hap1.G00000000042
SUPER_5	afgp17	partial	Hap1.G00000000043
SUPER_5	tryp3	complete	Hap1.G00000000044
SUPER_5	afgp/tlp2	partial	Hap1.G00000000045
SUPER_5	tomm40	complete	Hap1.G00000000046

**Hap2**			
**Chr**	**Gene name**	**Status**	**Gene ID**
SUPER_5	tryp1	complete	Hap2.G00000000001
SUPER_5	tryp1	complete	Hap2.G00000000002
SUPER_5	tryp1	complete	Hap2.G00000000003
SUPER_5	tryp1	complete	Hap2.G00000000004
SUPER_5	tryp1	complete	Hap2.G00000000005
SUPER_5	tryp1	complete	Hap2.G00000000006
SUPER_5	tryp1	partial	Hap2.G00000000007
SUPER_5	tryp1	complete	Hap2.G00000000008
SUPER_5	tryp1	partial	Hap2.G00000000009
SUPER_5	tryp1	complete	Hap2.G00000000010
SUPER_5	tryp1	partial	Hap2.G00000000011
SUPER_5	tryp1	complete	Hap2.G00000000012
SUPER_5	tryp3	complete	Hap2.G00000000013
SUPER_5	tlp	complete	Hap2.G00000000014
SUPER_5	afgp1	partial	Hap2.G00000000015

SUPER_5	afgp2	complete	Hap2.G00000000016
SUPER_5	tryp3	partial	Hap2.G00000000017
SUPER_5	tryp3	partial	Hap2.G00000000018
SUPER_5	afgp3	complete	Hap2.G00000000019
SUPER_5	afgp4	complete	Hap2.G00000000020
SUPER_5	tryp3	partial	Hap2.G00000000021
SUPER_5	tryp3	partial	Hap2.G00000000022
SUPER_5	afgp5	complete	Hap2.G00000000023
SUPER_5	tryp3	partial	Hap2.G00000000024
SUPER_5	afgp7	complete	Hap2.G00000000025
SUPER_5	afgp8	complete	Hap2.G00000000026
SUPER_5	tryp3	partial	Hap2.G00000000027
SUPER_5	tryp3	partial	Hap2.G00000000028
SUPER_5	afgp9	complete	Hap2.G00000000029
SUPER_5	afgp10	complete	Hap2.G00000000030
SUPER_5	tryp3	partial	Hap2.G00000000031
SUPER_5	tryp3	partial	Hap2.G00000000032
SUPER_5	afgp11	complete	Hap2.G00000000033
SUPER_5	afgp12	complete	Hap2.G00000000034
SUPER_5	tryp3	partial	Hap2.G00000000035
SUPER_5	tryp3	partial	Hap2.G00000000036
SUPER_5	afgp13	complete	Hap2.G00000000037
SUPER_5	afgp14	complete	Hap2.G00000000038
SUPER_5	tryp3	partial	Hap2.G00000000039
SUPER_5	afgp15	complete	Hap2.G00000000040
SUPER_5	afgp/tlp	complete	Hap2.G00000000041
SUPER_5	afgp16	complete	Hap2.G00000000042
SUPER_5	afgp17	partial	Hap2.G00000000043
SUPER_5	tryp3	complete	Hap2.G00000000044
SUPER_5	afgp/tlp	complete	Hap2.G00000000045
SUPER_5	tomm40	complete	Hap2.G00000000046
